# Beneficial Effects of Butyrate on Kidney Disease

**DOI:** 10.3390/nu17050772

**Published:** 2025-02-22

**Authors:** Tram N. Diep, Haoxin Liu, Liang-Jun Yan

**Affiliations:** Department of Pharmaceutical Sciences, UNT System College of Pharmacy, University of North Texas Health Science Center, Fort Worth, TX 76107, USA; tramdiep@my.unthsc.edu (T.N.D.); haoxinliu@my.unthsc.edu (H.L.)

**Keywords:** butyrate, gut microbiota, kidney disease, kidney injury, short-chain fatty acid, oxidative stress

## Abstract

The gut microbiota influences and contributes to kidney health and disease. Butyrate, a short-chain fatty acid molecule generated via the fermentation of gut bacterial catabolism of nondigestible dietary fiber, has been shown to exert numerous beneficial effects on kidney disorders. The objective of this review was to discuss the latest findings on the protective effects of butyrate on a variety of animal models of kidney injury. We conducted a PubMed search using the title word “butyrate” and keyword “kidney” to generate our literature review sources. The animal models covered in this review include ischemia–reperfusion renal injury, cisplatin- and folic acid-induced kidney injury, septic kidney injury, diabetic kidney disease (DKD), high-fat diet (HFD)-induced glomerulopathy, adenine-induced chronic kidney disease (CKD), high-salt-induced renal injury, and T-2 toxin-induced kidney injury in birds. The protective mechanisms of butyrate that are most shared among these animal model studies include antioxidative stress, anti-fibrosis, anti-inflammation, and anti-cell death. This review ends with suggestions for future studies on potential approaches that may modulate gut microbiota butyrate production for the well-being of kidneys with the kidney disorders covered in this review.

## 1. Introduction

The kidneys are essential organs in the body. They serve as the body’s filters by clearing and purifying the blood via eliminating toxins as waste [1]. The kidneys also participate in maintaining the blood acid–base balance and blood pressure, and serve as regulators of blood and bone health by secreting erythropoietin and vitamin D maturation [2]. Under starved conditions, the kidneys can also make glucose molecules via gluconeogenesis [3,4]. All these functions require ATP dependent active transport of molecules and ions in nephrons [5,6]. Indeed, each nephron contains many mitochondria because they have a high oxygen-consumption rate [7]. These unique characteristics of nephrons can also render the kidneys extremely vulnerable to insult and injury [6,8]. Therefore, there is clearly a need to identify and evaluate novel therapeutic agents that can target a variety of kidney diseases.

The gut microbiota plays a critical role in maintaining gut homeostasis, which can markedly affect the regulation of digestion and metabolism [9,10,11]. It is known that through the gut–kidney axis [12] a bidirectional communication occurs, which involves exchanges of metabolites and signaling molecules. This bidirectional relationship can impact the health of both the kidneys and the gut. Depending on a person’s physiological conditions, their use of dietary supplements, or pathophysiological conditions, alterations to the gut microbiota can either adversely or beneficially influence the functioning of the kidneys [13,14,15,16,17]. It has been demonstrated that the dysregulation of the gut microbiota can worsen kidney disease. This is primarily due to the disruption of intestinal epithelial cell function by microbiota-produced urinary toxins, such as phenyl sulfate [18]. These toxins, together with bacterial DNA, can then enter the circulation, further worsening kidney disease. One particular microbiota product is polysaccharides (LPSs), generated by Gram-negative bacteria, that can end up in the kidneys after intestinal barrier disruption due to dysbiosis and intestinal leakage. These LPSs can activate toll-like receptors and upregulate NF-κB, thereby prompting renal inflammation [12]. On the other hand, gut microbiota can also exert beneficial effects on kidney disease via the production of pro-health molecules, such as small-chain fatty acids including acetate, propionate, and butyrate [19,20]. In this review, we will focus on butyrate.

**Figure 1:**
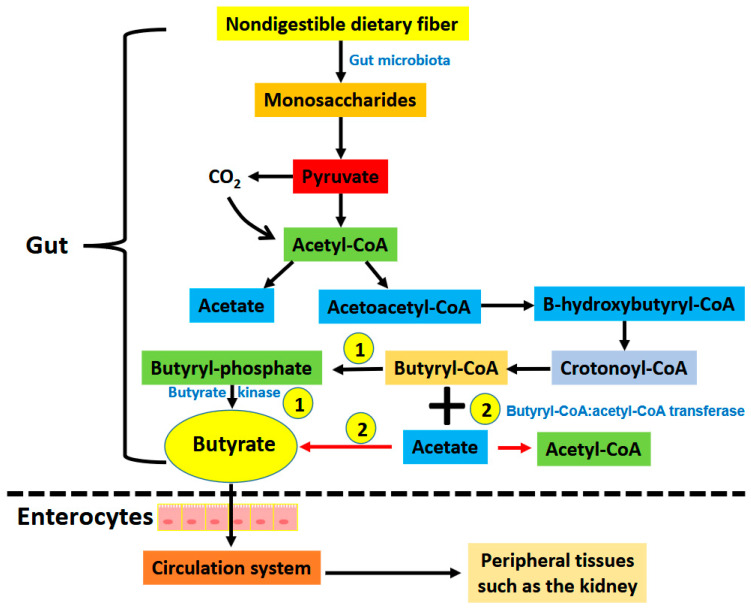
Pathways showing the generation of butyrate via gut bacterial fermentation of nondigestible dietary fiber (Please see text for details). There are two pathways that form butyrate in the gut. (1) Butyrate is formed from butyryl-CoA by the enzyme butyrate kinase. (2) The moiety of -CoA on butyryl-CoA is transferred to acetate forming acetyl-CoA and butyrate. This reaction is catalyzed by butyryl-CoA: acetyl-CoA transferase. Butyrate is then absorbed via butyrate transporters including monocarboxylate transporter and sodium coupled monocarboxylate transporters on the apical membrane of intestinal epithelial cells. This is followed by distribution of butyrate to other tissues including the kidneys and pancreas that have butyrate receptors such as GPR41, GPR43, and GPR 109A.

**Figure 2:**
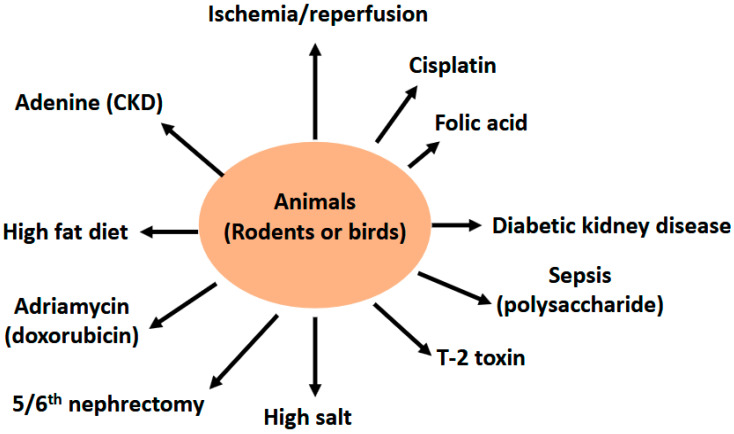
Diagram showing animal models of kidney injury discussed for the nephroprotective effects of butyrate in this article. These models are ischemia reperfusion injury, cisplatin- and folic acid-induced kidney injury, diabetic kidney disease (DKD) or diabetic nephropathy (DN), septic kidney injury induced by LPS, T-2 toxin-induced kidney injury in the bird, high salt kidney injury, 5/6th nephrectomy, adriamycin-induced kidney injury, high fat diet induced renal damage, and adenine-induced chronic kidney disease (CKD).

Butyrate can be derived directly from the diet (minor source) or endogenously from the gut bacteria via the fermentation of nondigestible dietary fiber (major source) [21,22,23]. The main bacteria that produce butyrate in the gut are *Coprococcus eutactus*, *Coprococcus comes*, *Coprococcus catus*, *Anaerostipes* spp., *Eubacterium hallii*, *Eubacterium rectale*, *Roseburia* spp., and *Faecalibacterium prausnitzii* [24]. It has been established that butyrate can be generated through two bacterial metabolic pathways in the gut (Figure 1). The first one involves the phosphorylation of butyrate-CoA to form butyryl-phosphate that is then converted to butyrate by butyrate kinase. The second pathway involves the transfer of the CoA functional group on the butyryl-CoA molecule to acetate with the concurrent formation of butyrate and acetyl-CoA via an enzyme known as butyryl-CoA: acetyl-CoA transferase [25,26]. Butyrate is then absorbed through the monocarboxylate transporters and sodium-coupled monocarboxylate transporters on the apical membrane of colon cells (also known as enterocytes) followed by their distribution to peripheral tissues, such as the brain, the liver, and the kidneys (Figure 1) [23,26]. The distribution of butyrate to these tissues is achieved by membrane receptors, such as GPR41, GPR43, and GPR109A [26]. It has been demonstrated that butyrate has antioxidant, anti-inflammation, and anti-fibrosis properties in a variety of organs and tissues [26,27,28,29,30,31]. In this review we discuss the renoprotective effects of butyrate on a variety of kidney diseases and kidney injuries, with a focus on experiments conducted on a variety of animal models (Figure 2). These animal models, as shown in Figure 2, include renal ischemia–reperfusion injury; cisplatin- and folic acid-induced AKI; DKD; septic kidney injury by LPSs; environmental toxins, such as T-2 toxin; high-salt-induced kidney injury; fifth/sixth nephrectomy, doxorubicin-induced kidney injury, high fat diet induced kidney injury, and adenine induced CKD. It should be noted that references cited in this review were derived from PubMed searches using the title word “butyrate” and the key word “kidney”. If a paper does not have the word “butyrate” in the title but contains both “butyrate” and “kidney” as key words, it was most likely excluded unless otherwise discussed.

While this review focuses on recent studies derived from animal models, clinical studies of the beneficial effects of butyrate on human kidneys have also been conducted in recent years. These studies often used sodium butyrate as a dietary supplement [25] and the patients recruited often had metabolic disorders such as obesity and diabetic kidney disease (DKD) or chronic kidney disease (CKD) [26]. These investigations have demonstrated that butyrate has a positive impact on human kidney disease in the context of metabolic disorders. For example, it has been found that patients with CKD showed decreased butyrate serum content and patients with DKD exhibited decreased butyrate levels in the serum [26]. Additionally, there was notable gut microbiota dysbiosis in DKD patients and serum butyrate concentrations were inversely correlated with DKD [26]. Insufficient butyrate due to dysbiosis was also found to contribute to recurrent kidney stone disease in humans [32]. These studies suggest that butyrate is a potential target for treating DKD or CKD. It should be noted that any positive effects of butyrate on other human kidney disorders have been less well evaluated.

## 2. Renoprotective Effects of Butyrate on Renal Injuries

### 2.1. Butyrate and Renal Ischemia—Reperfusion Injury

Renal ischemia reperfusion (RIR)-induced acute kidney injury (AKI) can be impacted by gut microbiota [33] and is a serious and frequent occurrence that can be caused by kidney transplantation, kidney surgery, vascular surgery and cardiac surgery [34,35]. AKI can occur in up to 35% of all hospitalized patients and is accompanied with a significant increase in mortality risk [36,37]. Non-fatal RIR-induced AKI, if untreated timely, could lead to development of chronic kidney disease and end stage renal failure [38,39,40], thereby increasing lengths of hospital stay, medical expenses, and mortality as well as physical and mental burden on the patients’ family members who are usually the caregivers [41,42].

In terms of the role of butyrate in renal ischemia reperfusion injury, it has been found that rats pre-treated with butyrate (300 mg/kg body weight, IV injection) followed by kidney ischemia reperfusion (30 min ischemia/24 h reperfusion) exhibited amelioration of kidney function and histological damage when compared with rats that were not pre-treated with sodium butyrate [43]. Butyrate pretreatment also attenuated neutrophil infiltration as reflected by a decreased myeloperoxidase activity. Furthermore, butyrate also attenuated tubular cell death by enhancing caspase-3 activation. On the other hand, TNF-α was decreased following butyrate pretreatment after ischemia reperfusion. In a separate study, Sun et al. further found that butyrate ameliorated renal ischemia reperfusion injury by mitigating oxidative stress and inflammation [44]. Another study by Wang et al. indicates that butyrate could ameliorate renal ischemia reperfusion injury by suppressing the expression of HES1 that further repressed the expression of PPARα by binding the PPARα promoter [45]. These studies elucidated that butyrate alleviated renal ischemia reperfusion injury via anti-oxidation and anti-inflammation mechanisms.

### 2.2. Butyrate and Cisplatin—Induced Kidney Injury

Cisplatin is a cancer treatment agent but can also cause kidney injury [46,47]. The major underlying mechanisms of cisplatin-induced kidney injury are thought to involve redox imbalance, oxidative stress, and mitochondrial dysfunction [47]. Chen et al. demonstrated that butyrate treatment significantly enhanced kidney function and decreased the magnitude of kidney fibrosis in the kidneys exposed to cisplatin [48]. These protective effects were associated with attenuated inflammatory response as reflected by decreased levels of kim-1, myeloperoxidase, NOX2, and TGF-β1, along with increased levels of IL-10. The authors further found that butyrate ameliorated cisplatin-induced gut microbiota dysbiosis. Favero et al. demonstrated that continuous butyrate treatment before cisplatin administration enhanced kidney resilience to acute kidney injury induced either by cisplatin or by folic acid [49]. The protective effect of butyrate was likely mediated by maintaining the expression of klotho, PGC-1α, and NLRP6 which otherwise were decreased by cisplatin induction of kidney injury. The renoprotective effect of butyrate on cisplatin induced kidney injury showed a dose-dependent manner [48].

### 2.3. Butyrate and Folic Acid—Induced Kidney Injury

Folic acid is also known as vitamin B9 that is implicated in 1-carbon metabolism which is crucial for cell survival and death [50]. While low dose folic acid is a nutrient and cofactor, high dose of folic acid is toxic to the kidney [50]. The major mechanisms by which folic acid induced kidney injury is obstruction of proximal tubules by crystallized folic acid, which can further cause redox imbalance, oxidative damage, impaired mitochondrial function, and increased fibroblast growth factor 23 [50]. Therefore, kidney injury in rodents can also be modeled by ingestion of high folic acid [50,51,52,53]. In a mouse model of folic acid-induced kidney injury, Corte-Iglesias et al. [54] demonstrated that butyrate injection decreased the content of renal injury markers, attenuated the expression of pro-inflammatory and pro-fibrotic markers as well as the loss of klotho. Butyrate was also found to slow down the progression from AKI to CKD in the folic acid model of kidney injury. This study agrees with the Favero study [49] whereby butyrate enhanced kidney resilience to folic acid induced injury.

### 2.4. Butyrate and Lipopolysaccharide (LPS)—Induced Kidney Injury

Sepsis is a leading cause of death in hospitalized patients, and acute kidney injury (AKI) is usually a severe consequence in such patients. While the mechanisms of sepsis-induced AKI are complex and remain to be elucidated, oxidative stress and mitochondrial dysfunction are thought to be implicated [55]. Septic kidney injury can be modeled in rodents by administration of LPS [56,57]. Hence, this animal model of kidney injury has been used to study the protective effects of butyrate on septic kidney injury. Dou et al. [58] found that butyrate could alleviate LPS-induced renal structural damage as reflected by decreased renal lesions and improved glomerular structure. Additionally, increased levels of creatinine, blood urea nitrogen, TNF-α, and IL-6 were decreased by butyrate treatment. Mechanistically, the authors demonstrated that the nephroprotective effect of butyrate was likely modulated by TLR2/4 that regulates rBD2 expression. Tian et al. [59] further found that butyrate could inhibit pyroptosis, thereby attenuating LPS induced kidney injury. Butyrate could also safeguard mitochondrial function to protect the kidney against septic renal injury [60].

### 2.5. Butyrate and Renal Failure Induced by 5/6th Nephrectomy (Nx)

The 5/6th nephrectomy animal model serves as an excellent platform for the study of renal failure [61,62,63]. This surgery removes one kidney and two thirds of the other kidney [64]. This robust kidney injury model has been shown to elevate serum creatinine levels severely when compared to that of control animals [64]. Gonzalez et al. [65] investigated the protective effect of butyrate on renal function using this animal model. In the absence of butyrate treatment, the Nx rats showed impaired insulin and glucose tolerance accompanied with increased gluconeogenesis in conjunction with decreased secretion of glucagon-like peptide-1 (GLP-1). The authors also noted a significant elevation in circulating LPS, indicating a leaky gut barrier. These parameters could be largely reversed by butyrate treatment, resulting in improved kidney function which was dependent on AMPK phosphorylation.

Curcumin, as a natural product that can enhance the production of butyrate in the gut, has been shown to ameliorate kidney injury in this animal model of kidney disease. Li et al. has reported that administration of curcumin could ameliorate renal fibrosis and attenuate renal inflammation in Nx rats. The authors further found that this protective effect of curcumin was likely due to the increased production of butyrate along with elevated levels of vitamin D [12].

### 2.6. Butyrate and Diabetic Kidney Disease (DKD)

DKD is a severe complication of diabetes mellitus [18,66] and can develop in approximately 35% of patients with type 1 or type 2 diabetes [6,67]. The hallmark of this chronic kidney disease is loss of podocytes, decrease in glomerular filtration rate, and proteinuria [6]. Therefore, DKD poses a significant risk factor for mortality in diabetic patients. While the underlying molecular mechanisms of DKD are complex, glucotoxicity-induced oxidative stress, redox imbalance, and mitochondrial abnormalities have been thought to be involved [6,68]. Butyrate has been demonstrated to be nephroprotective in DKD in a variety of rodent models of diabetes. One potential mechanism underlying butyrate nephroprotective effect on DKD involves histone butyrylation modification [69] and the inhibition of histone deacetylases (HDACs) [70], leading to amelioration of eNOS, iNOS, and TGFβ1-induced DNA damage, fibrosis and apoptosis. There are also other mechanisms by which butyrate protects against DKD, including: Nrf2 activation [71], amelioration of insulin resistance via modulation of intestinal permeability and mucin expression [65], inhibition of pyroptosis and apoptosis [72], mediation of the miR-7α-5p/p311/TGF-β pathway [73], and activation of AMPK/Sirt1/PGC-1α signaling pathway [74,75]. It has also been found that increased butyrate production in the gut from lactiplantibacillus plantarum NKK20 also contributes to DKD amelioration via the PI3K/Akt signaling pathway [76].

### 2.7. Butyrate and High Fat Diet (HFD) Induced Glomerulopathy

Obesity is a major public health issue. Its development is highly correlated with pathogenesis of chronic kidney disease [77]. It is known that glomerulopathy can be partly caused by overnutrition and obesity [78,79]. Using a mouse model of obesity-related glomerulopathy (ORG), Shi et al. [80] performed a study whereby 16 weeks’ feeding of HFD with the initiation of butyrate treatment at week 8 was conducted. The results indicate that butyrate alleviated renal injury, kidney oxidative stress, and mitochondrial abnormalities. The authors also found that butyrate could maintain mitochondrial structure and function that were mediated by the GPR43 and Sirt3 signaling pathway. When GRP43 and Sirt3 were inhibited, the protective effect of butyrate on ORG was largely abolished. This study again indicates that butyrate can directly modulate mitochondrial function to sustain normal kidney function. Additionally, ORG could also be alleviated by butyrate via its inhibitory effect on pyroptosis of glomerular endothelial cells.

### 2.8. Butyrate and Adenine—Induced Chronic Kidney Disease (CKD)

Chronic consumption of adenine can cause CKD by initiating chronic inflammation and renal fibrosis [81,82]. Using a CKD mouse model created by adenine administration, Tian et al. [83] found that butyrate content decreased as CKD progressed. When exogenous butyrate was supplemented to these adenine-treated mice, they found that renal fibrosis was attenuated, and the expression of those proteins associated with NLRP3-linked pyroptosis was suppressed. In vitro studies using cultured cells led to the discovery that the STING/NF-κB/p65 signaling pathway was mitigated by butyrate, and overexpression of STING could partially abolish the protective effect of butyrate in CKD. Therefore, butyrate can protect against CKD via an anti-inflammatory and anti-fibrosis mechanism. Gao et al. [84] also found that butyrate producing microbiota was decreased in CKD.

This adenine-induced CKD model has also been used to investigate the combined effect of resveratrol and butyrate on adenine-induced kidney injury exacerbated by microplastics [85]. The compound investigated by Huang et al. was called resveratrol butyrate esters (RBE), which enhanced the bioavailability of resveratrol and also exerted the biological effect of butyrate [85]. The authors found that microplastics exposure aggravated CKD-induced high blood pressure, and RBE treatment largely corrected the hypertension problem. Co-exposure of animals to microplastics and adenine led to nitric oxide deficiency, which was alleviated by RBE. They further found that RBS modulated both the classical and non-classical renin-angiotensin system to protect the kidney against injury. RBE was also found to alter gut microbiota composition, leading to increased butyrate content and GRP41 expression. Therefore, butyrate enhanced the renal protective effects of resveratrol in CKD.

### 2.9. Butyrate and Salt—Induced Hypertension and Renal Damage

Hypertension increases the risk for CKD [86,87]. It has been established that high dietary salt intake can contribute to the development of hypertension and CKD [88,89]. The deoxycorticosterone acetate (DOCA)/salt hypertensive rat model can be used to investigate the pathogenesis of hypertension and CKD and to evaluate the protective effects of a variety of compounds or natural products [90,91]. Using this model, Wu et al. [92] found that DOCA/salt treatment of uninephretomized rats markedly increased renal damage as reflected by increased kidney index, increased urinary albumin, elevated fibrosis and enhanced inflammation. When these hypertensive rats were treated with sodium butyrate, approximately 30% less salt-water intake and decreased Na^+^/Cl^−^ excretion in urine were observed. The authors further discovered that butyrate suppressed the protein content of several Na^+^ transporters stimulated by DOCA/salt. Moreover, mineralocorticoid receptor expression was down-regulated by butyrate, so was glucocorticoid-dependent protein kinase 1 (SGK1). Based on these findings, the authors concluded that butyrate might attenuate DOCA/salt-induced hypertension and kidney damage via inhibition of the MR/SGK1 signaling pathway.

### 2.10. Butyrate and Adriamycin—Induced Nephropathy

Adriamycin, also known as doxorubicin [93,94], is an antibiotic that is often used for cancer treatment [95,96]. Its clinical application is limited due to its cardiotoxicity and renal toxicity [97,98]. Hence, adriamycin can also be used to model kidney injury in rodents [99,100,101]. Felizardo et al. [102] tested the protective effect of butyrate on renal injury induced by adriamycin administered via tail injection. The authors focused their studies on G protein receptor (GPR) 109a using both wild type mice and GPR109a knockout mice. After adriamycin injection, the animals were treated with butyrate or butyrate-releasing high-amylose maize starch diet. The authors found that butyrate ameliorated proteinuria via preserving podocyte integrity and mitigated glomerulosclerosis and renal inflammation. The underlying protective mechanism was by enhancing podocyte-related proteins’ function and methylation and acetylation at the promoter sites of those genes that were essential for podocyte function. Moreover, the authors also found that the protective effect of butyrate was dependent on the expression of GPR109a. Therefore, butyrate’s protective role in adriamycin-induced nephropathy is modulated by GPR109a, which could also be enhanced by high dietary intake of butyrate or a prebiotic/probiotic diet. This study also demonstrates that butyrate is a life-saving agent as all adriamycin/butyrate-treated mice survived while only 20% of adriamycin-treated mice survived during the study.

### 2.11. Butyrate and Contrast—Induced Nephropathy

The kidney can also sustain damage induced by contrast media [103,104]. This form of kidney injury is often considered as acute kidney injury [105]. The underlying mechanisms of contrast induced nephropathy are thought to involve hypoxia or ischemia that impairs oxygen delivery to nephrons and mitochondrial function [106,107,108]. Reactive oxygen species (ROS) induced oxidative damage is also thought to be involved in contrast-induced nephropathy [109,110]. Using a rat model of contrast-induced renal injury, Machado et al. [111] demonstrated that butyrate was also nephroprotective against contrast-induced nephropathy. The authors found that not only were serum creatinine levels decreased by butyrate, but also the content of inflammatory markers such as IL-6 and NF-κB was mitigated. Moreover, the authors also found that elevated lipid peroxidation in contrast-induced kidney injury was also attenuated by butyrate. This study demonstrates that butyrate possesses antioxidant and anti-inflammation properties. It should be noted that the authors failed to detect a mitigating effect of butyrate on protein oxidation measured as protein carbonyl content that serves as a biomarker for protein oxidative damage [112,113,114]. The reason for this remains unknown.

### 2.12. Butyrate and T-2 Toxin Induced Renal Injury

Juvenile quails were utilized [115] by He et al. to evaluate the nephroprotective effects of butyrate on T-2 mycotoxin-induced renal toxicity. The authors found that the kidneys of these birds exhibited enhanced oxidative stress and inflammation as well as increased expression of nuclear xenobiotic receptors (NRXs), that altogether led to histopathological injury in the kidney. Such an injury could be mitigated by butyrate administration. Therefore, similar to those studies performed using rodent kidney injury models, butyrate also attenuates kidney injury in birds through anti-oxidation and anti-inflammation mechanisms.

## 3. Conclusions and Future Perspectives

**Figure 3:**
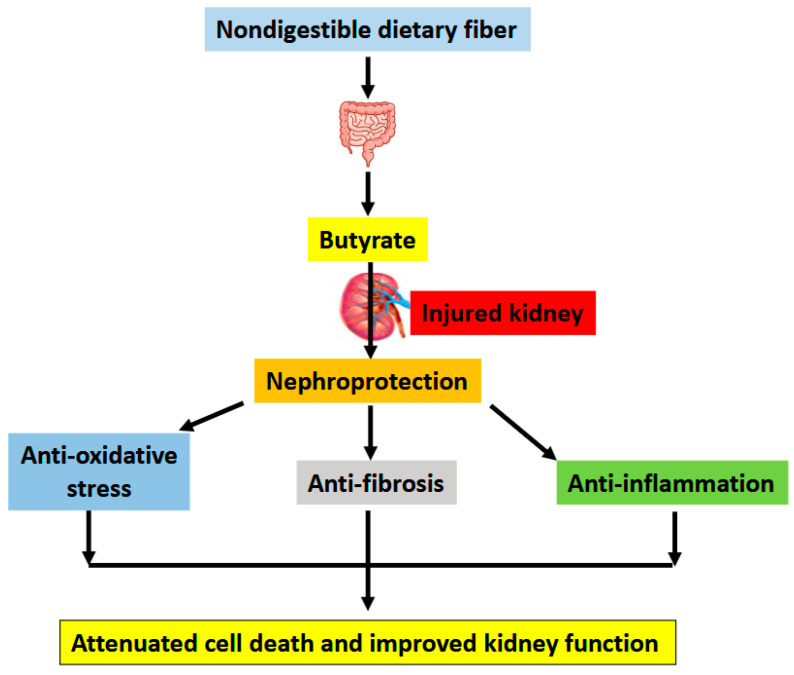
Major underlying mechanisms by which butyrate alleviates kidney injury. Butyrate is produced via fermentation by bacteria in the gut. This is followed by absorption by intestinal epithelial cells via butyrate transporters and distribution to the kidney via cellular butyrate receptors such as GPR41, GPR43, and GPR109A. Once inside nephrons, butyrate can exert its pro-survival functions including antioxidation, anti-fibrosis, and anti-inflammation, leading to attenuated cell death and improved kidney function.

Gut microbiota metabolites can contribute to kidney health and disease [9]. Which is indeed the case for butyrate, a short chain fatty acid molecule generated by gut bacterial fermentation of nondigestible dietary fiber. In this review, focusing on studies derived from animal models, we have discussed the beneficial effects of butyrate on a variety of kidney diseases. These animal models of kidney injury include ischemia-reperfusion injury, cisplatin- and folic acid-induced kidney injury, septic kidney injury modeled by LPS treatment, 5/6th nephrectomy-induced renal failure, DKD, HFD-induced glomerulopathy, adenine-induced CKD, salt-induced renal damage, doxorubicin-induced kidney toxicity, contrast media induced kidney injury, and T2-toxin-induced renal toxicity. The common underlying mechanisms of kidney injury in these animal models, largely involve oxidative stress, inflammation, fibrosis, apoptosis, pyroptosis, Nrf2 redox signaling pathway, and AMPK/sirtuin-PI3K/Akt signaling pathways. All these can be modulated by butyrate, mitigating kidney injury (Figure 3). One question arises from the mechanistic pathways shown in Figure 3 is which pathway elicited by butyrate is the most effective in terms of ameliorating kidney injury. Is it antioxidant, anti-inflammation, or anti-fibrosis? The answer is not a simple one. Perhaps, the antioxidant pathways elicited by butyrate are more unified than others, given that oxidative stress is often the unifying mechanism leading to cell death in numerous diseases [116].

Further studies investigating potential approaches that can enhance microbiota butyrate production in the gut are clearly warranted. These approaches, as shown in Table 1, all implicate dietary modulations of butyrate generation in the gut. We do not currently know which approach would be more powerful in terms of enhancing butyrate generation in the gut. It should also be pointed out that most current studies are limited to animal models. For clinical studies conducted in humans, randomized well-controlled investigations on a large scale are needed to evaluate positive effects of butyrate and butyrate-producing bacteria on a varity of human kidney diseases. In human studies, the dosage of butyrate as a supplement ranged from 200 mg/day to 4 g/day with varying durations being reported; and there was no negative effect of butyrate noted [25]. Whether higher dosages of butyrate with a long duration of treatment would produce some adverse effects remains to be investigated and a maximum safe dose of butyrate in humans also needs to be evaluated. Currently, there is no consensus regarding what type of dietary fibers are the most effective in stimulating butyrate production in the human gut. Moreover, how low salt intake impacts butyrate levels and renal damage in a positive way needs to be more comprehensively investigated. It is also not known how generalizable the effects of butyrate are on human gut microbiota. Finally, given that a dose-dependent renoprotective effect was observed in cisplatin-induced kidney injury [48], human studies involving various butyrate dosages should also be investigated in the future.

## Figures and Tables

**Table 1:** Potential approaches that may enhance gut microbiota production of butyrate for the benefit of kidney disease.

Approach	Reference
Consuming more fruits and vegetables or specific dietary fiber	[117,118,119,120]
Ingesting natural products, e.g., polysaccharides and polyphenols	[121,122]
Caloric restriction/dietary restriction	[7,123,124,125]
Ketogenic diet	[126,127]
Taking prebiotics or probiotics	[118,128]
Cutting back on salt	[129,130]
Exogenous sodium butyrate	[131,132]
